# Chemotherapy induced PRL3 expression promotes cancer growth via plasma membrane remodeling and specific alterations of caveolae-associated signaling

**DOI:** 10.1186/s12964-018-0264-8

**Published:** 2018-08-29

**Authors:** Balint Csoboz, Imre Gombos, Eniko Tatrai, Jozsef Tovari, Anna L. Kiss, Ibolya Horvath, Laszlo Vigh

**Affiliations:** 1 Institute of Biochemistry, Biological Research Centre of the Hungarian Academy of Sciences, Temesvari Krt. 62, Szeged, 6726 Hungary; 2 Department of Experimental Pharmacology, National Institute of Oncology, Budapest, 1094 Hungary; 3 Department of Anatomy, Histology and Embryology, Semmelweis University Budapest, Budapest, 1094 Hungary

**Keywords:** PRL3, Tumor therapy, Caveolae, Integrin, Rac1

## Abstract

**Background:**

The outcome of cancer therapy is greatly defined by the ability of a tumor cell to evade treatment and re-establish its bulk mass after medical interventions. Consequently, there is an urgent need for the characterization of molecules affecting tumor reoccurrence. The phosphatase of regenerating liver 3 (PRL3) protein was recently emerged among the targets that could affect such a phenomenon.

**Methods:**

The expression induction of PRL3 in melanoma cells treated with chemotherapeutic agents was assessed by western blotting. The effect of PRL3 expression on cancer growth was investigated both in vitro and in vivo. The association of PRL3 with the caveolae structures of the plasma membrane was analyzed by detergent free raft purification. The effect of PRL3 expression on the membrane organization was assayed by electron microscopy and by membrane biophysical measurements. Purification of the plasma membrane fraction and co-immunoprecipitation were used to evaluate the altered protein composition of the plasma membrane upon PRL3 expression.

**Results:**

Here, we identified PRL3 as a genotoxic stress-induced oncogene whose expression is significantly increased by the presence of classical antitumor therapeutics. Furthermore, we successfully connected the presence of this oncogene with increased tumor growth, which implies that tumor cells can utilize PRL3 effects as a survival strategy. We further demonstrated the molecular mechanism that is connected with the pro-growth action of PRL3, which is closely associated with its localization to the caveolae-type lipid raft compartment of the plasma membrane. In our study, PRL3 was associated with distinct changes in the plasma membrane structure and in the caveolar proteome, such as the dephosphorylation of integrin β1 at Thr788/Thr789 and the increased partitioning of Rac1 to the plasma membrane. These alterations at the plasma membrane were further associated with the elevation of cyclin D1 in the nucleus.

**Conclusions:**

This study identifies PRL3 as an oncogene upregulated in cancer cells upon exposure to anticancer therapeutics. Furthermore, this work contributes to the existing knowledge on PRL3 function by characterizing its association with the caveolae-like domains of the plasma membrane and their resident proteins.

**Electronic supplementary material:**

The online version of this article (10.1186/s12964-018-0264-8) contains supplementary material, which is available to authorized users.

## Background

Our understanding of cancer biology has rapidly expanded in the past decades, however, the number of approved tools that clinicians could use to treat cancer is still falling behind the complexity of the disease. Beside the emerging cancer treatments, conventional chemotherapy is still used on a daily basis. Chemotherapy can effectively induce the regression of cancers within a wide range of tumors, although it was shown to be considerably less effective in the prevention of tumor reoccurrence. The relapse of a cancer cell could represent a substantial threat to a patient as it can ultimately repopulate the original tumor site and give rise to distant metastases. Consequently, understanding and targeting the tumor survival strategies which counterbalance a chemotherapeutic treatment could supplement classical therapy and influence the outcome of the disease.

Among the targets that could affect such a phenomenon, the family of dual specificity phosphatases of regenerating liver (PRL) has received great attention in the recent years since their members, especially the phosphatase of regenerating liver 3 (PRL3), appeared to play a role in a wide range of cancerous processes [1, 2]. PRL3 was associated with primary tumor growth, tumor reoccurrence, and therapy resistance in many human cancers. Its expression was associated with increased tumor growth in primary gastric tumors [3]. This protein was also suggested as a marker to predict the relapse of gastric cancer [4] and invasive breast cancer [5]. It was further evaluated as a prognostic factor in hepatocellular carcinoma [6], ovarian [7], and colon cancer [8]. A role for PRL3 in tumor reoccurrence was also substantiated by a study targeting PRL3 by an antibody-based therapy, which was shown to be successful in preventing tumor reoccurrence and prolonging animal survival after surgical tumor removal [9]. Additionally, a chemical inhibitor of PRL3 was identified as a potent adjuvant to enhance the effectivity of cisplatin treatment in lung cancer cells [10], and the depletion of endogenous PRL3 was observed to sensitize acute myeloid leukemia cell to doxorubicin treatment [11].

Altogether, the above summarized findings strongly imply a role for PRL3 in a process that can ultimately contribute to tumor reoccurrence and therapy resistance. In our forthcoming experiments, we sought to delineate the so far vaguely described mechanism of PRL3 in this phenomenon, with the goal to characterize a possible tumor escape strategy against anticancer therapy.

## Methods

### Cell culture and treatments

B16F0, B16F1, and B16F10 (ATCC, Manassas, Virginia, USA; CRL-6322, CRL-6323, CRL-6475), mouse melanoma cells were maintained in RPMI (Thermo Fischer Scientific, Waltham, Massachusetts, USA); the media was supplemented with 10% FBS (Thermo Fischer Scientific). The cells were incubated in a humidified atmosphere containing 5% CO_2_ at 37 °C. Cells were treated at the indicated concentrations with doxorubicin, cisplatin, etoposide, and paclitaxel (all from Sigma Aldrich). Etoposide and paclitaxel were dissolved in DMSO. NSC23766 trihydrochloride (Sigma Aldrich, SML0952) was used to inhibit Rac1 function at the indicated concentration. In every experimental condition, except stated otherwise, the cells were treated with the chemicals for 24 h. All cell lines were routinely tested for mycoplasma contamination.

### Clone selection and stable expression of PRL3 in B16F0 cells

The human PRL3 cDNA was purchased from Origene (Rockville, Maryland, USA) in a pCMV6-XL5 vector. The PRL3 sequence was cloned into the EcoRI/XhoI sites of pcDNA 4/TO (Thermo Fischer Scientific). For inducible protein expression, pcDNA6/TR (Thermo Fischer Scientific) was used as a source of tetracycline repressor. B16F0 cells were transfected with pcDNA 4/TO or pcDNA4/TO-hPRL3 to achieve constitutive expression by using ExGen 500 (Thermo Fischer Scientific), according to the manufacturer’s instructions. In order to create clones with inducible protein expression, pcDNA6/TR and pcDNA4/TO-hPRL3 were cotransfected. Colonies were selected by the simultaneous addition of Zeocin at 700 μg/ml and Blasticidine at 8 μg/ml (Thermo Fischer Scientific), or Zeocin only. The expression of PRL3 was induced by the addition of doxycycline hyclate (Sigma Aldrich, D9891) to the cell culture media for 24 h (2 μg/ml).

### Western blot analysis

Cells were lysed in Laemmli buffer, and equal amounts of proteins were run on SDS/PAGE and transferred to a PVDF membrane. Membranes were probed with anti-PRL3 (Santa Cruz Biotechnology, Dallas, Texas, USA; 1:250, sc-130,355), anti-Cyclin D1 (Santa Cruz Biotechnology, 1:500, sc-8396), anti-Caveolin 1 (Thermo Fischer Scientific, 1:1000, 7C8), anti-Integrin ß1 (Abcam, Cambridge, UK; 1:2000, ab183666), anti-Integrin ß1 phospho T788/T789 (Abcam, 1:1000, ab5189), anti- GSK-3α/β (Cell Signaling Technology, Danvers, Massachusetts, USA; 1:1000, 5676) anti-phospho GSK3β Ser9 (R&D Systems Inc., Minneapolis, Minnesota, USA; 1:1000, AF1590), anti-Rac1 (EMD Millipore, Billerica, Massachusetts, USA; 1:1000, 05–389), anti-AKT (Cell Signaling Technology, 1:1000, 9272), anti-AKT phospho Ser473 (Cell Signaling Technology, 1:1000, 9271) and anti-GAPDH (Sigma Aldrich, 1:20000, G9545) antibodies. Data are representative of three independent experiments.

### Immunoprecipitation

Cells were lysed in RIPA buffer (10 mM Tris-Cl, 1 mM EDTA, 0.5 mM EGTA, 1% Triton X-100, 0.1% sodium deoxycholate, 0.1% SDS, 140 mM NaCl, pH 7.5). 500 μg of total proteins were used for each reaction. The lysates were precleared with Protein G Sepharose beads (GE healthcare Life Sciences, Chicago, Illinois, USA; 17,061,805) for 30 min. For the immunoprecipitation, anti-AKT (Cell Signaling Technology, 1:50, 9272) antibodies were mixed with the sample lysates, and the mixtures were incubated for 16 h at 4 °C. Subsequently, the immunocomplexes were incubated with the beads for 2 h at 4 °C. The beads were suspended in Laemmli buffer, and the immunoprecipitates were analyzed by western blotting. The coimmunoprecipitations were carried out in the same conditions, except that the cells were lysed in a low-detergent buffer (10 mM Tris-Cl, 1 mM EDTA, 0.5 mM EGTA, 0.5% Triton X-100, 0.1% sodium deoxycholate, 140 mM NaCl, pH 7.5), and anti-Rac1 (EMD Millipore, 1:50, 05–389) was used for the reaction.

### Rac1-GTP pulldown assay

Rac1-GTP detection was carried out with the commercial EZ-Detect Rac1 Activation Kit (Pierce, Waltham, Massachusetts, USA; 89856Y), according to the manufacturer’s instructions. From each sample, 20 μg of proteins were mixed with Laemmli buffer as a control for total Rac1 expression. An equal amount of 500 μg of proteins were transferred to glutathione beads containing 20 μg of GST-Pak1-PBD. The mixtures were incubated with gentle rocking for 1 h at 4 °C, then the beads were suspended in Laemmli buffer. The two fractions were analyzed by western blotting.

### Soft agar assay

For soft agar assays, 1 × 10^4^ of B16F0, B16F0-pcDNA4/TO, or B16F0-pcDNA4/TO-hPRL3 cells were seeded into a well of a 24-well plate in RPMI containing 0.7% agarose (Sigma Aldrich) and were propagated for 14 days. After the incubation period viable cells were measured by the resazurin dye-based alamar Blue assay (Thermo Fischer Scientific, DAL1025), according to the manufacturer’s instructions. Fluorescence was measured with a microplate fluorimeter (Thermo Fischer Scientific, Fluoroskan Ascent FL 5210450).

### Tumor growth assay

C57BL/6 J mice were obtained from the colony of the National Institute of Oncology, Budapest. Animal welfare and experimental procedures were performed in accordance with the related regulations of the ARRIVE guidelines [12] and with the animal welfare regulations of the host institute (permission number: 22.1/722/3/2010). For the tumor growth assay, subconfluent B16F0, B16F0-pcDNA4/TO, or B16F0-pcDNA4/TO-hPRL3 cells were harvested in PBS. 6 × 10^5^ cells was inoculated subcutaneously in a volume of 0.1 ml into the dorsal flanks of 8-week-old syngenic C57BL/6 J mice, as described previously [13]. Tumor size was measured three times a week with a caliper and expressed in mm^3^, using the formula for the volume of a prolate ellipsoid (length x width^2^ x π/6), as described previously [14].

### Plasma membrane isolation

The indicated cells were collected in TNM buffer (10 mM NaCl, 1.5 mM MgCl2, 10 mM Tris-HCl, pH 7.4) and were homogenized with the addition of glass beads (Sigma Aldrich, G4649) followed by a thorough vortexing. From this point, the isolation was carried out as described previously in Maeda et al. [15]. The interphase of each sample was collected into TNM buffer and centrifuged again at 100,000 g, at 4 °C for 1 h. The pellets were dissolved in Laemmli Buffer and their protein content was analyzed by western blotting.

### Detergent-free purification of caveolin-enriched membrane fractions

The indicated cells were scraped in sodium carbonate buffer (500 mM sodium carbonate, 25 mM 4-morpholineethane sulfonic acid, 150 mM NaCl, pH 11). Then, the cells were homogenized with the addition of glass beads to the cell suspension, followed by a thorough vortexing. From this point, the isolation was carried out as described previously in Song et al. [16]. For the western blot analysis of the resulting gradient, 1 ml fractions were collected from the top to the bottom.

### Confocal microscopy

Cells were fixed with 4% paraformaldehyde, permeabilized with Triton-X 100 (Sigma Aldrich), then incubated with anti-Rac1 (EMD Millipore, 1:100, 05–389), anti-PRL3 (Santa Cruz Biotechnology, 1:50, sc-130,355) or anti-cyclin D1 (Santa Cruz Biotechnology, sc-8396, 1:100, FITC conjugated) antibodies for 16 h at 4 °C. The Rac1 and PRL3 staining was followed by an incubation with anti-mouse IgG conjugated with Alexa555 (Thermo Fischer Scientific, 1:300, A31570) for 30 min at room temperature. Images were taken with a TCS SP5 laser scanning confocal microscope with Leica LAS software (Leica, Wetzlar, Germany), using a 63× oil-immersion objective. The nuclei were counterstained with 1 μg/ml 4,6-diamidino-2-phenylindole (Thermo Fischer Scientific, D1306).

### Imaging of di-4-ANNEPDHQ and calculation of general polarization (GP)

Cells were labelled with the plasma membrane-incorporating dye di-4-ANNEPDHQ (Thermo Fischer Scientific, D36802) at the concentration of 1 μM for 10 min. Image acquisition was carried out in a TCS SP5 laser scanning confocal microscope with Leica LAS software (Leica), using a 63× oil-immersion objective. An argon-ion laser at 488 nm was used for excitation, and the detection ranges of PMTs were set to 500–580 nm and 620–720 nm, respectively for the two emission channels. The GP values were calculated according to the following equation:

*GP* = (*I* _ (500 − 580) − *G I* _ (620 − 750))/(*I* _ (500 − 580) + *G I* _ (620 − 750)).

Where I represents the intensity of each pixel in the spectral channel (in nm) and G represents the calibration factor, which compensates the differences in the efficiency of collection in the two channels. In order to select the signal originating only from the plasma membrane we segmented our images with an image analysis software (CellProfiler, http://cellprofiler.org). The end product of this procedure was a plasma membrane segment which was originating from one cell. Each individual experimental group contained at least 80 membrane segments (80 cells) for one experiment. The measurements were repeated in three independent experiments.

### Electron microscopy

All procedures were carried out as described previously [17]. Briefly, the cells were fixed in 0.1 M cacodylate buffer containing 2% glutaraldehyde (Sigma Aldrich, G5882) (1 h, room temperature). The cells were pelleted in 12% gelatin, dehydrated with ethanol and contrast-stained stained with 1% uranyl acetate (Sigma Aldrich, 73,943) (1 h, room temperature), prior to araldite embedding. The samples were analyzed with a Hitachi H-7600 (Tokyo, Japan) transmission electron microscope.

### mRNA extraction and real-time reverse transcription-PCR

B16F0 cells were left untreated or treated with 2,5 μM doxorubicin. Total RNA was isolated 8 h after treatment with a NucleoSpin RNA kit (Macherey-Nagel, 740,955). Purified total RNA was subjected to spectrophotometry (260 nm and 280 nm) to assess purity and integrity. cDNA synthesis was carried out with a High-Capacity RNA-to-cDNA Kit (Thermo Fisher Scientific, 4,387,406). After cDNA synthesis PRL3 and GAPDH mRNA levels were determined by an end point PCR reaction. For the PCR reaction a Taq DNA polymerase was used (Thermo Fisher Scientific, EP0401) according to the manufacturer’s instructions. The following primers were used for the PCR reaction: PRL3 forward (5` GGACTCAGCCAGCTGTTTTT 3`), PRL3 reverse (5` CTTCCGCACCCCTAGAAATG 3`), GAPDH forward (5` AACGACCCCTTCATTGAC 3`), GAPDH reverse (5` TCCACGACATACTCAGCAC 3`).

## Results

### PRL3 protein expression is induced upon treatment with anticancer drugs

**Fig. 1:**
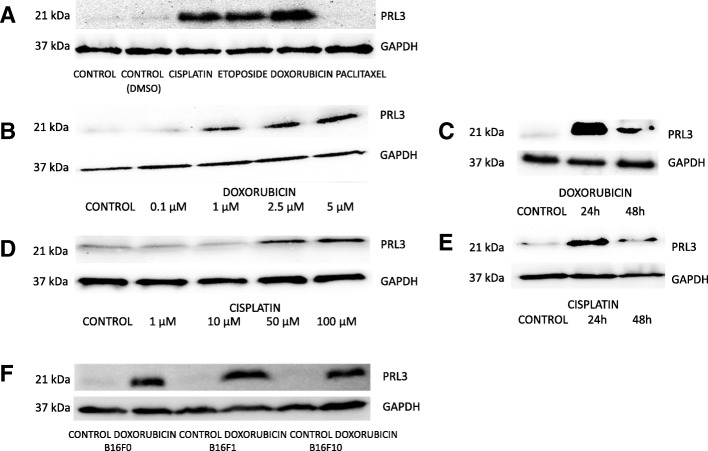
Induction of PRL3 expression by anticancer drugs in cancer cells. **a** PRL3 expression is upregulated in B16F0 cells upon treatment with various anticancer drugs. B16F0 cells were treated with 5 μM etoposide, 50 μM cisplatin, 2.5 μM doxorubicin, or 2 μM paclitaxel for 24 h. **b**, **d** Dose dependency of PRL3 induction upon doxorubicin and cisplatin administration. B16F0 cells were treated with the indicated amounts of drugs for 24 h. **c**, **e** Time dependency of PRL3 induction upon doxorubicin or cisplatin administration. B16F0 cells were treated with 2.5 μM doxorubicin or 50 μM cisplatin. PRL3 expression was analyzed 24 h after treatment or after a second 24 h incubation period without treatment. **f** PRL3 upregulation upon doxorubicin treatment is a characteristic feature of B16F0, B16F1, and B16F10 cells. The three B16 cell lines were treated with 2.5 μM doxorubicin for 24 h

Considering that key observations concerning the oncogenic action of PRL3 were made in B16 melanoma cells [18, 19], we chose to perform our studies in these cells by using the B16F0 line. In the light of the previously observed elevation of PRL3 expression in noncancerous MEF cells exposed to genotoxic carcinogens [20], we sought to find out if such phenomenon could be detected in B16F0 cells as well. In order to keep our study as clinically relevant as possible, we applied genotoxic stress conditions in the form of classical antitumor drugs that have a well-described effect on genomic DNA. Our studies revealed a distinct induction of the expression of PRL3 upon cell exposure to drugs such as doxorubicin, cisplatin, and etoposide (Fig. 1a). The application of paclitaxel, a drug that acts through a different mechanism by inhibiting microtubule organization, did not induce the expression of PRL3. The dose and time dependency of PRL3 induction in B16F0 cells was further examined upon treatment with doxorubicin or cisplatin as shown in Fig. 1b–e. The potential clinical importance of PRL3 induction is underlined by the fact that we administered the drugs close to their published IC50 values [21, 22]. Next, we sought to analyze if PRL3 induction occurs also in other cell lines. Therefore, other members of the B16 melanoma series were treated with the indicated amounts of doxorubicin. All three commercially available B16 cell lines, namely, B16F0, B16F1, and B16F10 showed a marked PRL3 expression upon treatment (Fig. 1f). These data are in line with a previous finding reporting the upregulation of PRL3 expression in cancer cells isolated from a mouse colon tumor that was treated with a DNA damaging carcinogen [23].

### PRL3 expression affects the tumorigenic properties of B16F0 cells

**Fig. 2:**
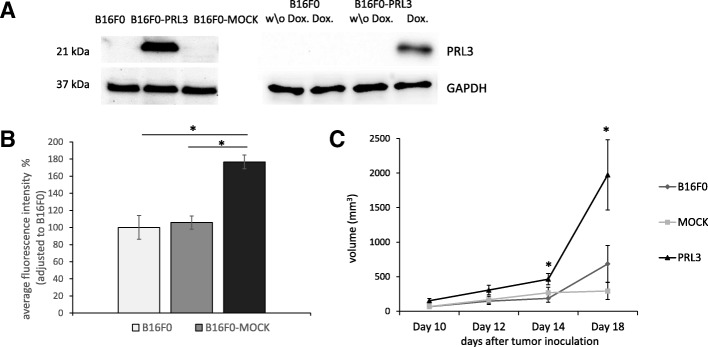
PRL3 contributes to tumorigenic growth. **a** PRL3 expression in B16F0 cells artificially expressing (human) PRL3, either in a constitutive or in a conditional (doxycycline-induced) manner. **b** Growth of B16F0 cells in anchorage-independent conditions. The indicated B16F0 cells were seeded into agar-containing media and cultured for 14 days. Colony formation from single cells was assessed by measuring cell viability with a resazurin-based Alamar blue assay (*n* = 3, SEM). * *p* ≤ 0.005 tested by Student t-test. **c** Tumor volume changes of subcutaneously injected B16F0 cells in mice. The indicated cells were injected subcutaneously into C57BL/6 J mice (*n* = 6, SEM). * *p* ≤ 0.05 tested by Aspin-Welch t-test. The volumes of the tumors were measured at the indicated time points

In order to assess the possible benefits conferred by the induction of PRL3 in cancer cells, we made an attempt to model this phenomenon by the artificial expression of (human) PRL3 in B16F0 cells. To this end, we created cell lines expressing PRL3 under either a constitutive or an inducible promoter, whose induction was provided by a tetracycline-controlled expression system (Fig. 2a). At first, we analyzed the effect of PRL3 expression on anchorage-independent growth by measuring the ability of these cells to establish three-dimensional colonies from a single cell in a soft agar assay. Our results revealed a significant increase in colony growth upon PRL3 expression in B16F0 cells constitutively expressing the phosphatase (Fig. 2b). Next, we aimed to test the validity of these data in an in vivo setting to evaluate primary tumor growth. To this aim, we injected the same set of cells subcutaneously into mice. The data we gained from these experiments showed that the PRL3-expressing B16F0 cells established subcutaneous tumors larger than those formed by control cells (Fig. 2c). This result is in line with other observations in the literature indicating that the disruption of the mouse PRL3 gene reduced colon tumor formation in carcinogen-treated mice [23], and that PRL3 expression was associated with tumor initiation and self-renewal in colorectal carcinoma [24]. Furthermore, nonphysiological PRL3 expression was shown to induce stem cell-like signatures in leukemia cells and corresponded with the presence of leukemia-initiating cells in the population [25]. Therefore, it is plausible that PRL3 contributes to tumor initiation or reinitiation, thereby influencing the outcome and relapse rate of cancer.

### PRL3 associates with the plasma membrane and localizes to the caveolae-rich detergent-resistant fraction

**Fig. 3:**
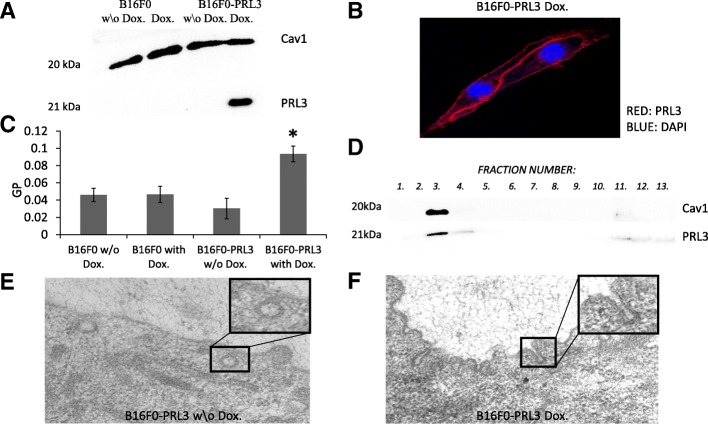
PRL3 affects the structure of the plasma membrane and localizes to caveolae. In all experiments, PRL3 expression was induced by the addition of doxycycline to the culture media for 24 h. **a**, **b** Analysis of PRL3 localization by plasma membrane isolation or by confocal imaging. **c** GP value changes upon PRL3 expression. Membrane order was assessed by the addition of the membrane incorporating dye di-4-ANNEPDHQ. Data is expressed as the mean of the GP values of at least 80 plasma membrane segments (SEM). * *p* < 0.001 tested by Student t-test. **d** PRL3 localizes to the Caveolin 1-rich submembrane fraction in doxycycline-induced B16F0-PRL3 cells **e**, **f** Representative TEM images of the membrane of B16F0-PRL3 cells with or without doxycycline induction: **e** non-induced B16F0-PRL3 cells show an intact caveolae morphology (resolution: 15000X), **f** PRL3-expressing B16F0 cells show elongated caveolae-like membrane formations (resolution: 15000X). Caveolae-like structures are indicated with arrows

Considering that our data connected PRL3 to a distinct phenotype that might promote tumor growth, we sought to decipher the functional background of PRL3 action. First, we analyzed the membrane localization of PRL3, since it seems crucial for PRL3 oncogenic function [18]. Our results confirmed PRL3 membrane localization in B16F0 cells by the analysis of isolated plasma membrane fractions and by confocal imaging (Fig. 3a, b and Additional file 1: Figure S3). Next, we concentrated our efforts on evaluating the nature and membrane environment of this interaction. First, we analyzed the general plasma membrane structure by the addition of the membrane incorporating dye di-4-ANEPPDHQ which, upon intercalation into the lipid bilayer changes its emission spectra according to the lipid packing order of the membrane. The normalized ratio of intensity of the two emission spectra of the probe gives the value of the generalized polarization (GP), which provides a measure of membrane order in the range between + 1 (gel) and − 1 (liquid-crystalline). According to our results, PRL3 expression changed the overall plasma membrane structure towards a more ordered, rigid state (Fig. 3c). This data indicates a PRL3-mediated effect on membrane organization and suggests that the PRL3-associated oncogenic phenotype could be better defined through a deeper analysis of the membrane. For this reason, we focused on determining the submembrane localization of PRL3. Our experiments revealed that the detergent-resistant, caveolae-type membrane microdomain region of the plasma membrane was a primary localization site for PRL3 (Fig. 3d). In order to validate our lipid raft fractionation method we have also analyzed the distribution of a non-raft protein, HSC70 within the same experimental conditions (Additional file 1: Figure S4.). To verify the presence of intact caveolae in our cells, we further analyzed caveolae morphology in B16F0 cells by transmission electron microscopy. The detection of the classical flask-shaped caveolae structures at the membrane of B16F0 cells (Fig. 3e) further support the localization of PRL3 in caveolae. However, it is necessary to note that in the PRL3-expressing cells we observed unusual elongated, tubular caveolae, different from the classical flask-shaped structures (Fig. 3f).

### The presence of PRL3 at the plasma membrane alters the caveolae-resident proteome

**Fig. 4:**
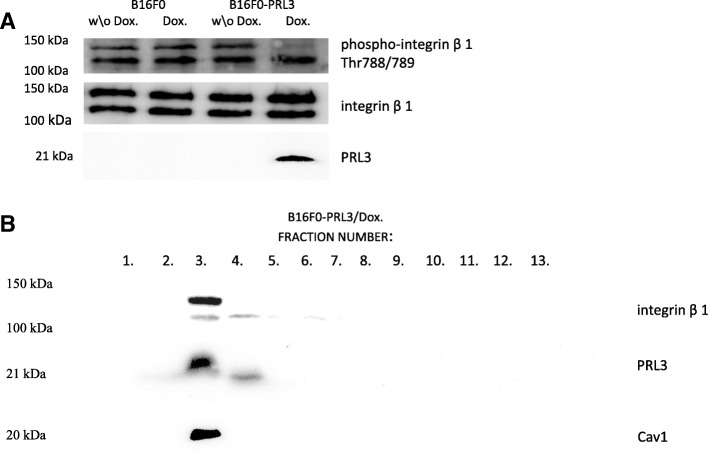
PRL3 expression affects integrin ß1 phosphorylation (**a**) Integrin ß1 shows dephosphorylation at Thr788/789 upon the induction of PRL3 expression with doxycycline. (**b**) Raft fractions corresponding to the caveolae region of the membrane from doxycycline-induced B16F0-PRL3 cells were analyzed for the presence of integrin ß1. Only the 150 KDa form of integrin ß1 is present in the caveolae membrane fraction and it cofractionates with PRL3

Based on the knowledge that the action of PRL3 is connected to the membrane and especially to the caveolae subfraction of the membrane, we concentrated our efforts on characterizing the alterations in caveolae in terms of their associated proteome upon PRL3 expression. We were especially interested in identifying possible substrates for PRL3 that could explain the observed oncogenic effect of the phosphatase in B16F0 cells. We particularly focused on integrin receptors, since integrin function is partly regulated in caveolae, and PRL3 was described in previous studies to interact with integrins and affect their expression and localization [26, 27]. Moreover, integrins are described to be central in adhesion-associated cell growth signaling [28]. Previously PRL3 was described to dephosphorylate the 783 tyrosine of integrin β1 [27] although, we were able to detect any phosphorylation at this site in our cells (data not shown). However, our experiments revealed that integrin ß1 was dephosphorylated at its Thr788/789 site upon PRL3 expression (Fig. 4a). Western blot experiments revealed a dual signal for integrin ß1, approximately at 100 and 150 KDa, similar to a previous observation which associated this phenomenon with different glycosylation patterns of the protein [29]. We could observe dephosphorylation only of the 150 KDa form of integrin ß1 (Fig. 4a). However, our raft fractionation experiments revealed that only the 150 KDa form of the integrin ß1 was associated with caveolae in our system. The colocalization of integrin ß1 and PRL3 in the plasma membrane was further verified by detecting both proteins in the same caveolae, membrane raft fraction (Fig. 4b). The Thr788/789 site of integrin ß1 has been attributed a dual function, as the phosphorylation of this site was shown to be necessary for the assembly of adhesion complexes, whereas its dephosphorylation appeared to be required for cell cycle progression [30, 31]. The studies describing these functions also implicated the phosphatase PP2A, which catalyzes the dephosphorylation of integrin ß1 at Thr788/789 promoting cell cycle progression [31]. Considering that the phenotype we observed in B16F0 cells could result from the dephosphorylation of ß1 at Thr788/789, we further analyzed the downstream effectors of PP2A in the hope to find possible common players interacting with both this phosphatase and PRL3. The studies describing the cell cycle regulatory role of PP2A identified Rac1 as a key effector of PP2A [31]. Therefore, we decided to analyze Rac1 function in B16F0 cells upon PRL3 expression.

**Fig. 5:**
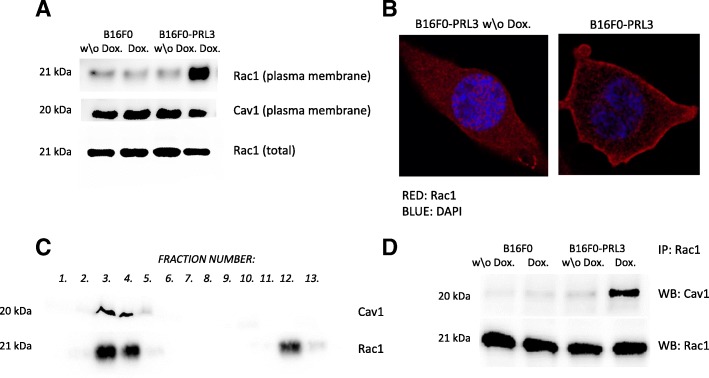
PRL3 expression affects the translocation of Rac1 to the plasma membrane. (**a**) Western blot analysis of isolated total membranes from the indicated cells shows that PRL3 expression results in enhanced translocation of Rac1 to the plasma membrane. (**b**) Representative confocal images of Rac1 plasma membrane localization in PRL3-expressing cells. **c** Rac1 localizes to the caveolae fraction in the doxycycline-induced B16F0-PRL3 cells. (**d**) Rac1 coimmunoprecipitation with the major caveolae scaffold Caveolin 1 indicates that Rac1 translocate to the caveolae fraction upon PRL3 expression

Since Rac1 membrane localization is crucial for its activation and for the subsequent activation of the signaling pathways regulating cellular growth, we analyzed Rac1 subcellular distribution by confocal imaging and by comparing the ratio of Rac1 in plasma membrane isolates (Fig. 5a and b). As our results identified an increased Rac1 plasma membrane targeting upon expression of PRL3, we further analyzed Rac1 association with submembrane regions. Considering the localization of PRL3 to the caveolae region of the membrane, we concentrated specifically on these membrane domains. We demonstrated that Rac1 associated with caveolae in PRL3-expressing cells by analyzing its membrane distribution by a sucrose gradient-based fractionation method (Fig. 5c). In addition, we determined the degree of association of Rac1 with caveolae by immunoprecipitation, assessing the ratio between Caveolin1 and Rac1 in a protein complex resulting from direct protein–protein interactions. The result of these experiments showed an increased direct physical interaction between Caveolin 1 and Rac1 in the presence of PRL3, which suggests that Rac1 translocated specifically to the caveolae regions of the membrane (Fig. 5d). Since increased Rac1 membrane targeting was already shown to positively affect cell growth [32], it is plausible that it could be responsible for the proliferation advantage of the PRL3-expressing B16F0 cells.

### Caveolar proteome changes induce downstream signaling associated with the regulation of cellular growth

**Fig. 6:**
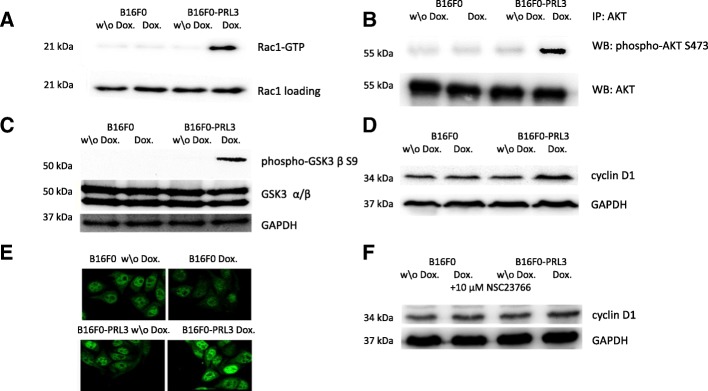
PRL3 expression affects cellular growth-associated signaling (**a**) Rac1-GTP loading increases upon PRL3 expression. **b** AKT phosphorylation increases at Ser473 upon PRL3 expression. **c** GSK phosphorylation increases at Ser9 upon PRL3 expression. **d** Cyclin D1 levels are upregulated upon PRL3 expression. **e** Representative confocal images of the localization of cyclin D1 in the indicated cells. Cyclin D1 localizes to the nucleus. **f** Rac1 inhibition decreases the elevated cyclin D1 levels. The indicated cells were treated with 10 μM NSC23766 for 24 h in order to inhibit Rac1-dependent elevation of cyclin D1 levels

In order to characterize the signaling downstream of Rac1 which could be accountable for the observed phenotype, we relied on previously established observations describing Rac1-associated signaling in the context of growth regulation. Rac1 GTP-binding is a prerequisite for Rac1-dependent regulation of the cell cycle. Therefore, our aim was to analyze if this event occurred in our system upon PRL3 expression. Our data clearly indicated a higher proportion of GTP-bound Rac1 (Fig. 6a) in doxycycline-treated B16F0-PRL3 cells, compared to control cells. As such modulation of signaling was further associated with increased phosphorylation of AKT kinase on the activator site Ser473 in the context of cellular growth, we analyzed the phosphorylation state of AKT. Our data revealed AKT phosphorylation at Ser473 upon PRL3 expression, which indicates the activation of AKT (Fig. 6b). AKT participates in anchorage-dependent growth by phosphorylating GSK kinase at its Ser9 residue, thereby inactivating it. In doxycycline-induced B16F0-PRL3 cells, we observed a strong phosphorylation of GSK at Ser9 (Fig. 6c). GSK is directly responsible for the regulation of the cell cycle by fine-tuning the protein amounts of cyclin D1. This results from GSK-dependent phosphorylation of cyclin D1 at its Thr286 residue, which targets it to degradation. Thus, we further analyzed the amount and localization of cyclin D1 upon PRL3 expression. As shown in Fig. 6d and in Additional file 1: Figure S5., we detected an increased amount of cyclin D1 in B16F0 cells upon PRL3 expression compared to control cells; the localization of cyclin D1 in the nucleus was verified by immunofluorescence staining (Fig. 6e). In order to clarify the role of Rac1 in the observed phenomena, we inhibited Rac1 chemically by the water-soluble NSC23766 inhibitor [33]. This molecule was described to inhibit the activation of Rac1 by interfering with the interaction of Rac1 and its GEFs without any effect on other Rho GTPases [34]. The addition of the Rac1 inhibitor to PRL3-expressing B16F0 cells resulted in the drop of cyclin D1 levels (Fig. 6f). The remaining slight increase might result from an incomplete Rac1 inhibition from the inhibitor molecule. Our preliminary experiments showed a certain decrease in the basal control levels of cyclin D1 in the presence of the inhibitor NSC23766 (data not shown) which has also been described by Liu et al. [35]. Therefore, we were consciously using a lower concentration of the inhibitor in order to avoid the over inhibition Rac1 which might have led to false data. Nonetheless, we do not rule out the idea that Rac1 might not be the only effector in the membrane which is responsible for the increased cyclin D1 levels.

## Discussion

Hereby, we are the first to describe an upregulation of PRL3 expression in cancer cells upon exposure to genotoxic chemotherapeutic agents. The fact that elevated PRL3 levels were already observed upon carcinogen treatment in other cell lines [23, 36, 37] suggests that PRL3 upregulation upon chemotherapy could also occur in a wide range of cancers. Our results could also be important in explaining previous observations that doxorubicin was more effective in PRL3-silenced leukemia cells [11], and the results of a drug screen showing that a chemical inhibitor of PRL3 strengthened the antitumor effect of cisplatin [10]. According to our data, these phenomenon might rely on the ability of these drugs to influence the expression of PRL3.

We were able to identify an increase in PRL3 mRNA levels upon doxorubicine treatment in B16F0 cells, which suggests that the observed elevation at the protein level might originates from a direct transcriptional upregulation (Additional file 1: Figure S1). Our in vivo tumor growth data supports this hypothesis, as PRL3-expressing cells were able to establish larger tumors when injected subcutaneously into mice.

The PRL3 responsiveness of a cancer cell could rely on a unique genetic background acquired by the cells during oncogenesis. We have assayed for PRL3 expression induction upon doxorubicin treatment in a few other cell lines as the A375 human melanoma and the SHSY5Y human neuroblastoma cells (data not shown) which have shown to have a certain level of PRL3 mRNA expression [36] but we could not recapitulate the phenomena in these cells. We think that the B16 cells represent a model where their unique genomic rearrangements allow the PRL3 protein to show such an induction profile. The work of Basak et al. [20] highlights the importance of the p19^Arf^ protein as a regulator of cell arrest in association with PRL3 expression in MEF cells. Another study which analyzes the genomic background of the B16 melanomas revealed the loss of the p19^Arf^ due to a large genomic deletion in these cells [38]. This study also shows the presence and nucleolar localization of P53 in the B16 cells. This particular arrangement might give the basis for the phenomena we observed in our work.

Previously, excessive PRL3 expression was associated with cell cycle stop in a noncancerous cells [20]. Therefore, it is plausible that, within normal conditions, upstream regulators are present in the cells, which could be responsible to keep the effects of PRL3 at bay. These counterbalances might be lost during tumor development. The fact that PRL3 was associated with the initiation of colorectal tumors upon a harsh carcinogenic treatment further suggests that PRL3 might be specifically present in tumors with large-scale genomic mutations [23, 24]. As PRL3 was previously detected as a P53 target gene [20], and there is a detectable level of P53 in the B16 cells [38], it is plausible that this transcription factor is responsible for the observed PRL3 induction in our studies. In our experiments the expression levels of the PRL3 protein correlated with the induction of P53 however, they did not overlapped fully (Additional file 1: Figure S2). PRL3 shows an earlier induction profile than P53 however, the peak of PRL3 induction is overlapping with the peak of the P53 levels. Since the promoter of PRL3 was described to contain other putative binding elements for stress-related transcription factors, such as NF-κB and STAT3 [39], we hypothesize that P53 is not the sole transcription factor which determines the levels of PRL3 under (genotoxic) stress conditions.

Our observations also suggest that PRL3 may act in a similar way as PP2A, which was described to regulate the cell cycle through the dephosphorylation of integrin ß1 at Thr788/789 [30]. It is tempting to speculate that this site serves as a switch for regulating adhesion-dependent cell growth. By modulating this phosphorylation site, phosphatases like PRL3 can serve as fast-acting switches on integrin receptors and subsequently regulate integrin-associated signaling.

Rac1 was shown to be a mediator of integrin-mediated cell cycle regulation, however the exact mechanism of its membrane recruitment and the specific guanine nucleotide exchange factors (GEFs) that could regulate its activation have not been fully understood yet [40]. Based on our data, we suggest that the caveolar membrane compartment can serve as a location for the transmission of signals by integrins through Rac1 in the context of cell growth regulation. Rac1 could control many downstream signaling processes depending on its interaction with regulatory elements. In Rac1-mediated migration, Rac1 recruitment to the focal adhesion points is initiated by the membrane adaptor p130Cas and integrin ß3, which allows the interaction between Rac1 and a GEF, i.e., Dock180 [41]. However, Dock180-mediated control over Rac1 signaling is associated only with cell migration, and it is not known what GEFs regulate Rac1-mediated cell cycle signaling. It is plausible that the dephosphorylation of integrin ß1 at Thr788/789 by PP2A or by PRL3 could act as a recruiting signal towards Rac1. A specific, as yet not known GEF in the caveolar compartment could be implicated in cell cycle regulation. The partitioning of Rac1 into caveolae could define its signaling outcome by spatially restricting its submembrane localization and subsequently determining its interaction with caveolae-associated regulatory factors.

It is noteworthy that the amount of caveolae in the membrane decreases during cancer progression [42], therefore caveolae might be present in early-stage cancers but not in more advanced tumors. The reason why PRL3 is connected to such a wide variety of signaling pathways in different cancer cells [43] might rely on the fact that PRL3 is not in its original subcellular environment in those tumors where caveolae are absent. Therefore, considering the wide, possible substrate variety of PRL3 nonspecific interactions could account for the diverse actions observed for PRL3. The hypothesis that PRL3 exerts a different role depending on the existence of caveolar structures is further strengthened by the observation that in primary uveal melanoma cells PRL3 affects integrin clustering predominantly at the focal adhesion sites of the membrane [44]. Uveal melanoma cells, however, express a considerably lower, or no amounts of Caveolin 1 compared to melanoma cells of cutaneous origins [45], such as B16F0 cells.

Moreover, the structural membrane changes that we observed during PRL3 expression might contribute to the step-by-step progression of cancer cells towards a more aggressive phenotype. Membrane raft reorganization upon stress were already connected with gene expression changes and stress resistance in B16 cells [46, 47]. Conditions, such as mechanical stretching or osmotic swelling, are associated with the disassembly of caveolae [48]. Therefore, it is tempting to speculate that the increased membrane rigidity in the PRL3-expressing cells might result in a similar cellular response in the long term. Such a loss of caveolae from early-stage cancer cells could consequentially contribute to cancer progression [49]. This hypothesis is substantiated by our morphological observations describing elongated, unnatural caveolae structures in the PRL3-expressing cells.

In summary, we identified PRL3 as a protein upregulated in cancer cells upon treatment with several clinically relevant antitumor therapeutics. In our melanoma model, PRL3 expression was associated with enhanced cancer growth in parallel with increased cyclin D1 levels. The elevation of cyclin D1 was shown to be associated with the dephosphorylation of integrin ß1 at Thr788/789 by PRL3 and the attraction of Rac1 to the plasma membrane. Therefore, here we propose that the genotoxic stress-mediated induction of PRL3 could rewire caveolae signaling, which can be critical in tumor initiation and relapse. In conclusion, targeting PRL3 in parallel with chemotherapy might prove useful for the therapy of PRL3-expressing cancers.

## Conclusions

Our results deliver the first evidence of the upregulation of PRL3 expression in cancer cells upon exposure to anticancer therapeutics. The presence of this oncogene in the plasma membrane and the subsequent alterations in the plasma membrane structure could serve as identifying factors for tumor recurrence. In addition, our study also highlights the importance of targeting PRL3 in parallel with chemotherapy to prevent the relapse of PRL3-expressing cancers.

## Additional file

Additional file 1: **Figure S1.** Elevation of PRL3 transcript levels upon doxorubicin treatment. **Figure S2.** Detection of elevated P53 protein levels upon doxorubicin treatment. **Figure S3.** Detection of actin in the isolated plasma membrane fraction. **Figure S4.** Detection of HSC70 fractionation within the plasma membrane. **Figure S5.** Densitometric analyses of cyclin D1 elevation upon PRL3 expression. (PDF 431 kb)

## Abbreviations

GEF: Guanine nucleotide exchange factor
GP: General polarization
GSK: Glycogen synthase kinase
PP2A: Protein phosphatase A2
PRL3: Phosphatase of regenerating liver 3
Rac1: Ras related C3 botulinum toxin substrate 1
