# Supplementary material for: Chemotherapy induced PRL3 expression promotes cancer growth via plasma membrane remodeling and specific alterations of caveolae-associated signaling
Source: Cell Commun Signal. 2018 Aug 29;16:51. doi: 10.1186/s12964-018-0264-8 (PMC6116440; doi:10.1186/s12964-018-0264-8)
Supplement: Supplementary file 1 — Figure S1. Elevation of PRL3 transcript levels upon doxorubicin treatment. Figure S2. Detection of elevated P53 protein levels upon doxorubicin treatment. Figure S3. Detection of actin in the isolated plasma membrane fraction. Figure S4. Detection of HSC70 fractionation within the plasma membrane. Figure S5. Densitometric analyses of cyclin D1 elevation upon PRL3 expression. (PDF 431 kb) [file 12964_2018_264_MOESM1_ESM.pdf]

Supplemental Data

Chemotherapy induced PRL3 expression promotes cancer growth via plasma membrane remodeling and specific alterations of caveolae-associated signaling

Balint Csoboz; Imre Gombos; Eniko Tatrai; Jozsef Tovari; Anna L. Kiss; Ibolya Horvath; Laszlo Vigh

Figure S1. Elevation of PRL3 transcript levels upon doxorubicin treatment.

We were able to detect an elevation in the PRL3 mRNA levels upon doxorubicin administration, which suggests that the increased PRL3 protein levels are originating from a direct transcriptional upregulation of the gene. Although, considering the previous work of Wang et al. [1] which highlights an important inhibiting interaction between the 5′-UTR region of the PRL-3 mRNA with one of the members of the hnRNP family of RNA- and DNA-binding proteins, the poly (C)-binding protein 1 (PCBP1), we do not rule out the possibility that the PCBP1 inhibition on the translation is altered during stress conditions. However, the fact that PCBP1 could not inhibit completely PRL3 translation [1] suggests that this mechanism could serve only as a fine-tuning switch in the regulation of cellular PRL3 levels.

Figure S2. Detection of elevated P53 protein levels upon doxorubicin treatment.

B16F0 cells were left untreated or treated with doxorubicin at the outlined concentration for 24 hours. Two samples were harvested after this point (24h) one sample were incubated for another 24 hours (48h). P53, PRL3 and GAPDH levels were determined by western blotting as described in the materials and methods section. For the detection of P53 we used the following antibody: Cell Signaling Technology, 1:1000, 2524.

Figure S3. Detection of actin in the isolated plasma membrane fraction.

A plasma membrane fractionation experiment similar to Figure 3A, which analyses the presence of actin in the isolates, is included to serve as a negative control. Plasma membrane was isolated from the B16F0 or B16F0-PRL3 cells either treated with doxycycline (Dox.) or left untreated (w\o Dox.) as outlined in the materials and methods section. 20-20 μg of proteins were loaded to an SDS polyacrylamide gel from the plasma membrane isolates and from the same set of total cell isolates. Actin expression was analyzed by Western blotting as described in the materials and methods section with the following antibody: Cell Signaling Technology, 1:1000, 4967.

Figure S4. Detection of HSC70 fractionation within the plasma membrane.

A raft fractionation experiment similar to Figure 3D, which shows the distribution of the HSC70 protein within the plasma membrane, is included to serve as a negative control. The HSC70 does not copurifies with lipid rafts but fractionates to the non-raft fraction of the plasma membrane

which is rich in phosphatidylserine [2]. Our figure shows that the majority of the HSC70 protein is associated with the later membrane fractions.

The plasma membrane fractions of B16F0 cells were isolated as outlined in the materials and methods section. The distribution of the HSC70 protein between the fractions were determined by Western blotting as described in the materials and methods section, with the following antibody: StressMarq, 1:2000, SMC-104.

Figure S5. Densitometric analyses of cyclin D1 elevation upon PRL3 expression

Cyclin D1 expression differences were quantitated by the densitometric analysis. The measured cyclin D1 values were normalized to the corresponding GAPDH values. All data was compared to the B16F0 cells without doxycycline treatment (B16F0 w/o Dox.). The data is expressed as percentage differences.

References

1. Wang H, Vardy LA, Tan CP, Loo JM, Guo K, Li J, et al. PCBP1 Suppresses the Translation of Metastasis-Associated PRL-3 Phosphatase. Cancer Cell. 2010; 18:52–62.

2. Vega VL, Rodríguez-Silva M, Frey T, Gehrmann M, Diaz JC, Steinem C, et al. Hsp70 translocates into the plasma membrane after stress and is released into the extracellular environment in a membrane-associated form that activates macrophages. J Immunol. 2008; 180:4299–307.

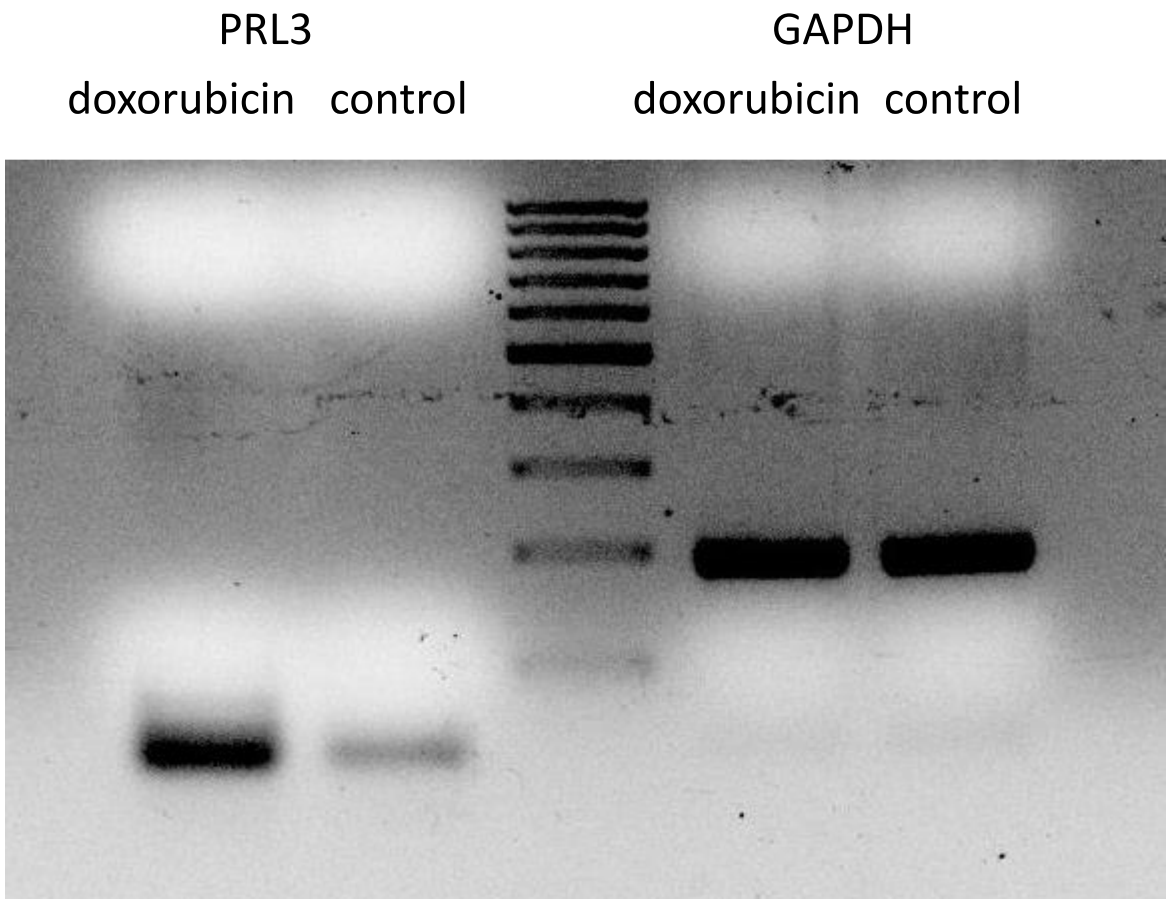

Figure S1.

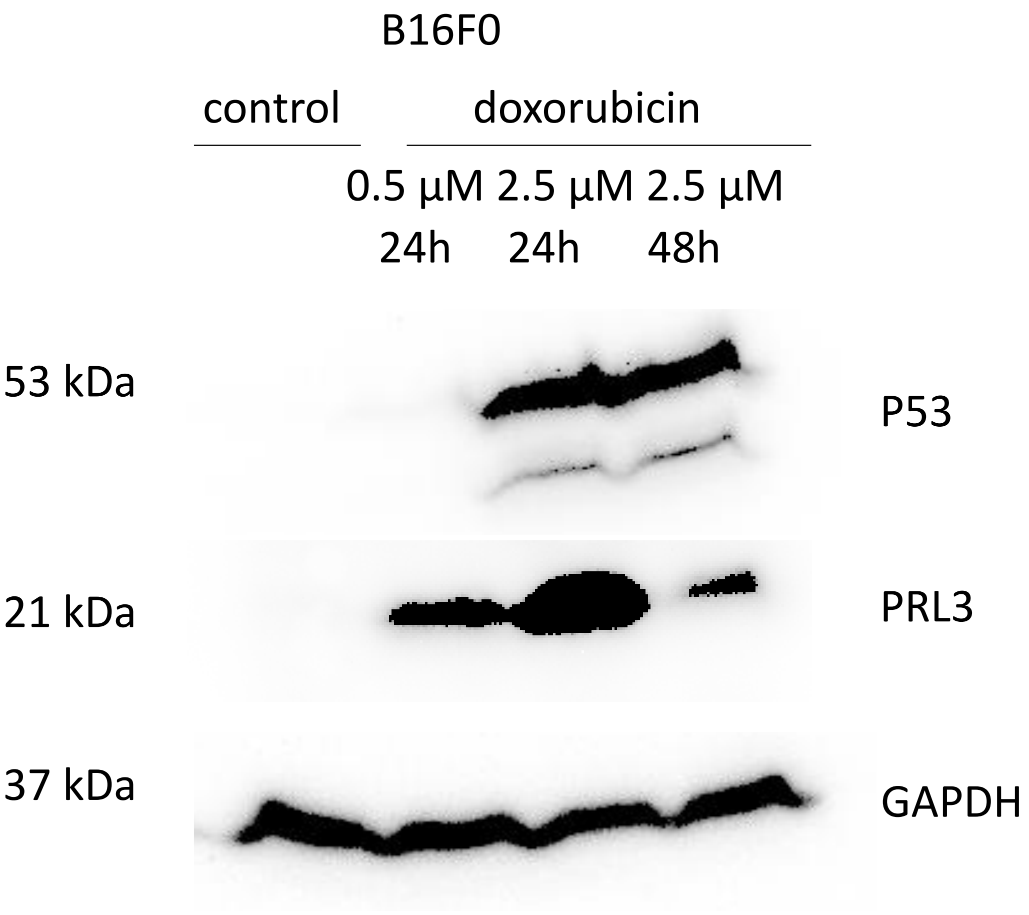
B16F0
control
doxorubicin
0.5 µM 2.5 µM 2.5 µM
24h 24h 48h
53 kDa
P53
21 kDa
PRL3
37 kDa
GAPDH

Figure S2.

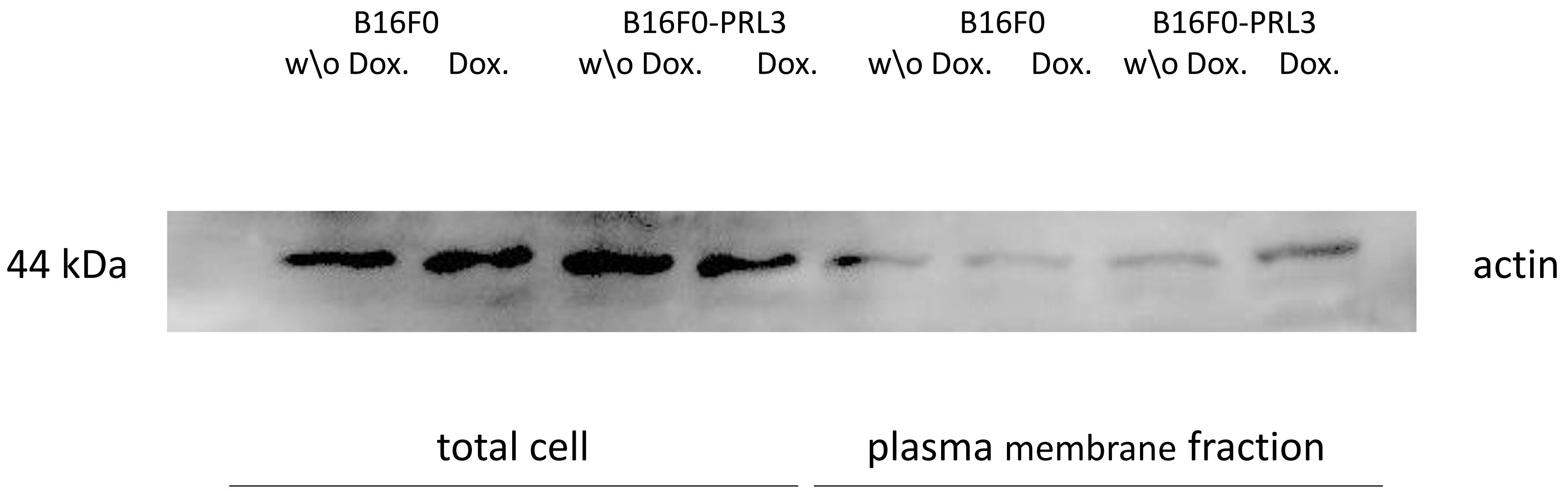
B16F0
B16F0-PRL3
B16F0
B16F0-PRL3
w\o Dox.
Dox.
w\o Dox.
Dox.
w\o Dox.
Dox.
w\o Dox.
Dox.
44 kDa
actin
total cell
plasma membrane fraction

Figure S3.

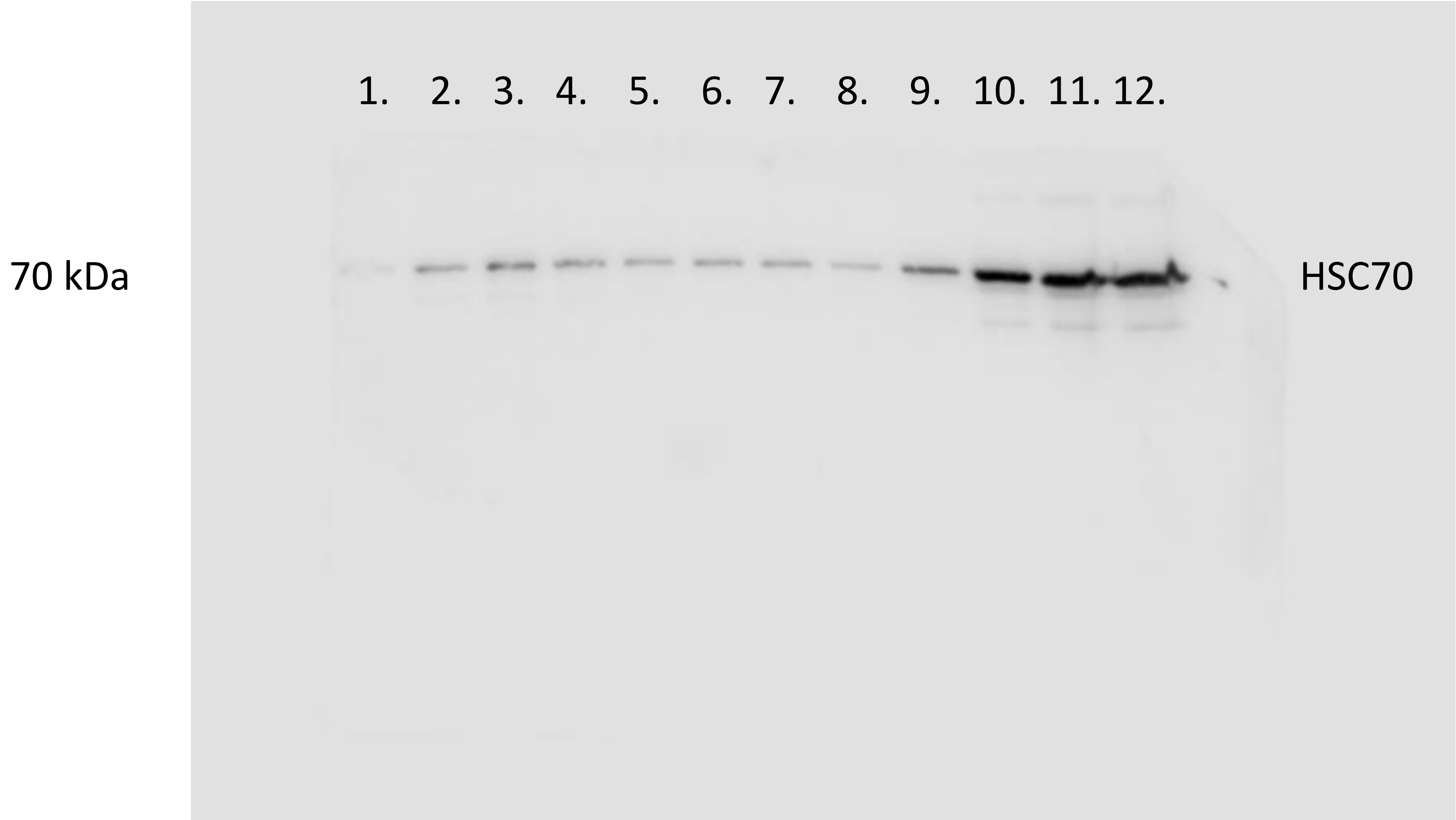

Figure S4.

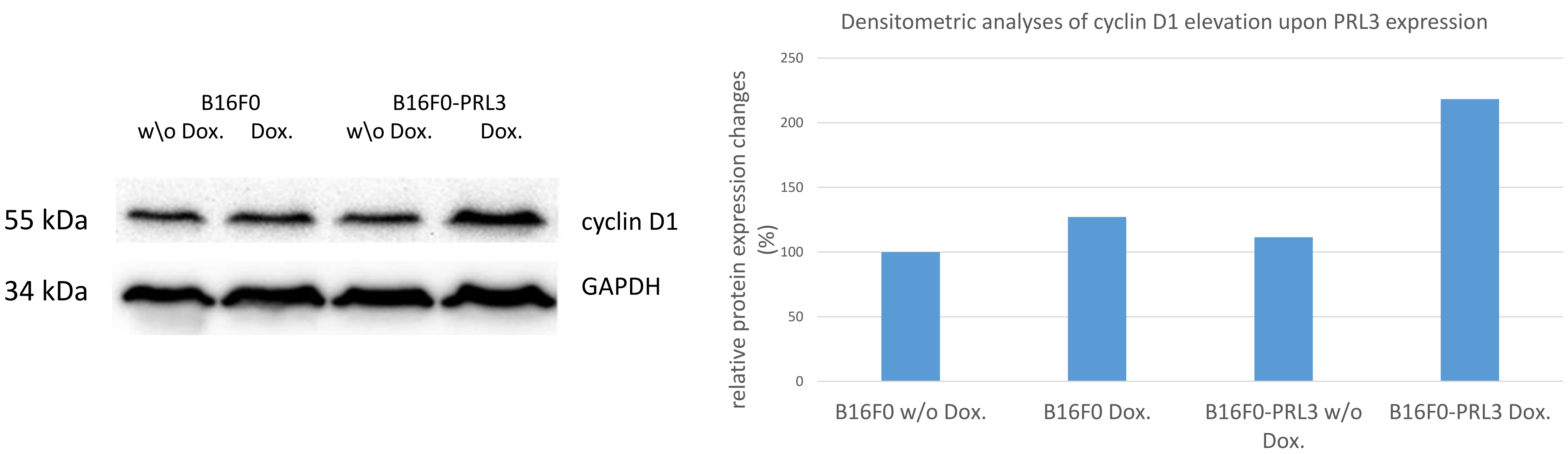
B16F0
B16F0-PRL3
w\o Dox.
Dox.
w\o Dox.
Dox.
55 kDa
34 kDa
cyclin D1
GAPDH
Densitometric analyses of cyclin D1 elevation upon PRL3 expression
relative protein expression changes (%)
250
200
150
100
50
0
B16F0 w/o Dox.
B16F0 Dox.
B16F0-PRL3 w/o Dox.
B16F0-PRL3 Dox.

Figure S5.
